# Mass Spectrometry-Based Multivariate Proteomic Tests for Prediction of Outcomes on Immune Checkpoint Blockade Therapy: The Modern Analytical Approach

**DOI:** 10.3390/ijms21030838

**Published:** 2020-01-28

**Authors:** Julia Grigorieva, Senait Asmellash, Lelia Net, Maxim Tsypin, Heinrich Roder, Joanna Roder

**Affiliations:** Biodesix, Inc, 2970 Wilderness Place, Boulder, CO 80301, USA; senait.asmellash@biodesix.com (S.A.); lelia.net@biodesix.com (L.N.); maxim.tsypin@biodesix.com (M.T.); heinrich.roder@biodesix.com (H.R.); joanna.roder@biodesix.com (J.R.)

**Keywords:** Biomarkers, immune checkpoint inhibitors, multivariate tests, circulating proteome, mass spectrometry

## Abstract

The remarkable success of immune checkpoint inhibitors (ICIs) has given hope of cure for some patients with advanced cancer; however, the fraction of responding patients is 15–35%, depending on tumor type, and the proportion of durable responses is even smaller. Identification of biomarkers with strong predictive potential remains a priority. Until now most of the efforts were focused on biomarkers associated with the assumed mechanism of action of ICIs, such as levels of expression of programmed death-ligand 1 (PD-L1) and mutation load in tumor tissue, as a proxy of immunogenicity; however, their performance is unsatisfactory. Several assays designed to capture the complexity of the disease by measuring the immune response in tumor microenvironment show promise but still need validation in independent studies. The circulating proteome contains an additional layer of information characterizing tumor–host interactions that can be integrated into multivariate tests using modern machine learning techniques. Here we describe several validated serum-based proteomic tests and their utility in the context of ICIs. We discuss test performances, demonstrate their independence from currently used biomarkers, and discuss various aspects of associated biological mechanisms. We propose that serum-based multivariate proteomic tests add a missing piece to the puzzle of predicting benefit from ICIs.

## 1. Introduction

Cancer immunotherapy, a treatment that selectively augments a patient’s anti-tumor immune response, constitutes the major breakthrough in oncology of the recent decades, for the first time introducing the possibility of curative treatment in advanced disease. The introduction of immune checkpoint inhibitors (ICI) has resulted in a substantial proportion of patients (15–35%, depending on cancer type) experiencing durable benefit, with overall survival curves seeming to plateau after three years with low risk of death thereafter [1,2]. However, up to 85% of patients do not respond to these agents, while being exposed to significant toxicity from treatment [3]. The need for reliable biomarkers of clinical outcomes and for treatment monitoring has been appreciated from the beginning of the immune checkpoint blockade era. In this paper we provide a brief review of the current status of biomarker research in the field. Further, we discuss several mass spectrometry-based proteomic tests that are currently in clinical development. These tests were created to assist in optimizing treatment with immune checkpoint inhibitors and are different from existing biomarkers because, instead of focusing on the properties of the tumor or tumor microenvironment (TME), they utilize the circulating proteome as a source of information about the host response to the disease on the whole-organism, systemic level. Since changes in the blood during cancer development and progression arise from contributions from tumor cells, tumor microenvironment, and the host [4], the circulating proteome provides essential cues about the processes determining the response to immunotherapy. Here we discuss some basic principles underlying the development of multivariate proteomic tests and illustrate their implementation using previously validated results for three different tests based on a modern, machine learning approach; we also discuss the performance of an established proteomic test VeriStrat^®^ in the context of immunotherapy. Further we use set enrichment analysis methods [5] to determine the association of the multivariate tests with various biological processes of interest, and finally we gauge the potential implications of these results for clinical applications and research.

To date, the most extensively studied ICI biomarkers are those based on the understanding of the mechanism of action of the immune checkpoint inhibition in the tumor, i.e., activation of the adaptive immune response that is present but was peripherally suppressed. Expression of PD-L1 on tumor cells and in the tumor micro-environment was, naturally, the first biomarker candidate for anti-PD1 therapy. It has shown predictive value in several, but not all, clinical studies and is currently used as a biomarker in some indications, despite known shortcomings in its performance. These shortcomings are related to various technical and biological factors, such as variability in tissue collection timing, the antibody and methodology used for staining, the heterogeneity and dynamics of PD-L1 expression within different tumors [6]. The non-straightforward effect of PD-L1 expression on tumor-infiltrating lymphocytes and other immune cells versus the malignant cell population in response to treatment complicates the picture. As a result, while improved survival is often correlated with increased PD-L1 expression, some patients with high PD-L1 have poor outcomes, while patients with low or undetectable expression may derive benefit from ICI therapy [7,8].

Interest in tumor mutational burden (TMB) as a biomarker was predicated on a concept that more mutations yield more T cell–recognized tumor neoantigens, potentially resulting in stronger antitumor immune responses [9]. TMB was first shown to be associated with response to cytotoxic T lymphocyte antigen-4 blockade in patients with melanoma; subsequently, correlations with outcomes in patients with non-small cell lung cancer (NSCLC) treated with anti–PD-1 therapy (pembrolizumab) and in other solid tumor types [10] were demonstrated. Microsatellite instability (MSI) and deficient DNA mismatch repair (dMMR) are examples of defects in DNA repair that result in the accumulation of very high levels of TMB. Using measurements of MSI and dMMR as a marker for drug response, the US Food and Drug Administration (FDA), for the first time, granted approval for cancer treatment based on a common biomarker rather than the site of tumor origin (e.g., nivolumab was approved for treatment of advanced line metastatic cancer and pembrolizumab was approved as a therapy for adult and pediatric patients with unresectable or metastatic solid tumors with high MSI or dMMR [11,12]). However, TMB alone does not represent direct evidence of tumor immunogenicity and has serious limitations as a biomarker because local anti-tumor cytotoxicity does not directly correlate with neoantigen abundance [13]. Recent studies found no correlations between TMB and the efficacy of ICIs in combination with chemotherapy in NSCLC [14], diminishing the clinical utility of TMB for patient selection. It is increasingly clear that while some mutations result in ‘higher quality’ antigens, which are more readily identified as ‘non-self’ by the immune system and are more likely to induce a robust antitumor immune response, others are less likely to be recognized by the immune system [9].

The complexity of tumor biology and its interaction with the immune system suggests that studying individual components is unlikely to adequately predict clinical outcomes in response to immune-targeted therapy [15]. Characterization of the TME by the abundance, location, and phenotype of tumor infiltrating lymphocytes and other immune cells provides a more comprehensive picture of the various interactions between the tumor and the host. Several assays, most notably, Immunoscore^®^ (HalioDx, Marseille, France) for colon cancer [16], have been developed to characterize “immune contexture” and have shown utility as a prognostic tool, independent of treatment. However, while Immunoscore has demonstrated superiority to gold standard TNM classification, its adoption in routine clinical practice has been slow, and predictive performance with respect to specific immunotherapies has not yet been established [17]. Further efforts have been aimed at combining several biomarkers that, due to their independent nature, together may capture different aspects of neoantigenicity and T cell activation and may provide greater predictive and prognostic relevance than each of them individually [18,19]. For example, in a study utilizing whole-exome and RNA sequencing information from the Cancer Genome Atlas, a linear combination of three variables—CD8+ T-cell abundance, TMB, and high PD1 gene expression—resulted in a higher correlation with response to anti–PD-1/PD-L1 therapy across cancer types than the individual components [20]. 

However, despite some success in improving biomarker performance, the observed patient response to treatment is quite often contrary to biomarker-based predictions. It is apparent that there is some other level of biological information, possibly related to more general aspects of the organism’s reaction to the disease, that is not captured by existing tests. One of the reasons may be that immune response in the tumor microenvironment, while being a crucial factor defining tumor fate and therapy responses [21] and a necessary condition for immune checkpoint blockade efficacy, is not sufficient. The host response on the level of the whole organism also plays a significant role. Indeed, tumor–host interactions extend well beyond the local tissue microenvironment and tumors not only respond to, but also actively perturb host systems distantly [22]. These perturbations result in changes in peripheral blood leukocyte activity and in alterations of the circulating proteome. To date, cell-based surrogate markers for systemic inflammation, such as a neutrophil-to-lymphocyte ratio (NLR) measured in circulation, are most studied and have shown significant prognostic value in cancer [23,24]. An index combining the derived neutrophil/leukocyte-lymphocyte ratio (dNLR) with lactate dehydrogenase (LDH) levels demonstrated strong prognostic potential in NSCLC in different clinical modalities, including immune checkpoint inhibitors [25]. Nonetheless, the predictive potential of these measurements for ICIs relative to other therapies has not been established.

Serum/plasma provides a rich source of biological information with a dynamic range of protein abundance exceeding 10 orders of magnitude [26]. Around 24% of classical plasma proteins are associated with immune response [27]. They reflect various aspects of the functioning of adaptive and innate immune systems, as well as other related physiological processes, including wound healing, extracellular matrix remodeling, angiogenesis, complement cascades, and metabolic changes [4,28]. Wide-ranging data has been accumulated on the influence of circulating innate immunity components, such as acute-phase reaction and complement activation, on the natural course of disease and the response to treatment, modulated by direct effects on tumor cells and by supporting a cancer-abetting microenvironment [29,30].

We propose a simultaneous measurement of multiple components of the circulating proteome and their combination in a multivariate classifier using a nonlinear, multivariate model in a hypothesis-independent fashion. This approach does not limit the resulting test to a pre-specified mechanism of action and may integrate general information on the state of the host better than existing biomarkers, adding a missing piece to the puzzle of predicting response to immune therapy. The challenge is to find a way to extract this information and convert it into a clinically useful, accessible, and validated tests.

## 2. Multivariate Serum-Based Tests: Challenges and Solutions

Despite major investments by both academia and industry and a lot of progress in data analysis, very few multivariate biomarkers have been adopted in clinical practice. The reasons for this are of both a fundamental and methodological nature: On the one hand, while some discovered biomarkers have been independently validated, they provided only incremental and not sufficiently strong predictions to be deemed useful in broad practice; on the other hand, many proposed biomarkers turned out to be false discoveries, showing a significant effect in the test development stage, but failing in independent validation studies. In other words, they were overfitted [31,32]. The problem of overfitting is often related to straightforward application of unsuitable algorithms to small sample sets that are characterized by many variables. It is estimated that, for example, a supervised deep-learning algorithm would need around 5000 labeled training examples to achieve acceptable performance [33], while the number of samples available for training from a typical clinical study rarely exceeds a couple of hundreds, and often is less. To overcome this difficulty, one has to employ an approach to test development that is suitable for a particular setting and will minimize the risk of overfitting, such as that described in detail in Roder et al. [34,35]. For example, appropriate regularization of the chosen machine learning method is critical to minimize risk of overfitting (regularization techniques in machine learning can minimize overfitting, typically via the addition of noise, supplementary information, or controlling the complexity of the model). While regularization can be achieved quite well using some traditional approaches, such as Random Forest [36], it can be useful to apply the regularization concept of dropout [37] that is typically employed in modern deep learning [34,35]. As development cohorts are often small, isolation of a “hold out” test set (i.e., subset of samples withheld from use in training) that is population representative and large enough to provide a good assessment of test performance is often impossible. However, careful use of an ensemble average over multiple splits of the development set into training and test sets (“bagging”) [38] allows reliable test classifications to be obtained for all samples in the development cohort, via “out-of-bag” estimators [39]. This permits a robust assessment of test performance to be made on the test development cohort with the simultaneous use of data from all samples in the cohort [34,35].

Another important aspect of developing clinically meaningful tests is obtaining an appropriate set of samples that represents the target population. As was mentioned above, availability of the samples, and therefore, the sample set size, is crucial. Additionally, the type of samples (e.g., blood vs. tissue vs. urine vs. spinal fluid) should contain maximum information pertinent to the problem. A growing body of evidence demonstrates that cancer therapy itself induces host-mediated and systemic responses, which influence the delicate balance within TME and affect tumor progression [40]. From this standpoint, plasma and serum have certain advantages: They are readily available and, unlike biopsies, can be obtained more often and through less invasive procedures, and constitute an abundant source of dynamic information about the state of the organism. 

Matrix-Assisted Laser Desorption/Ionization (MALDI) Time of Flight (TOF) mass spectrometry is a suitable tool for developing proteomic tests, as it provides a high throughput platform for the reproducible measurement of circulating proteins and peptides [41]. Although in its standard application it is limited by relatively low sensitivity, it is possible to vastly increase the information content of MALDI-TOF spectra by applying Deep MALDI^®^ technology, which dramatically increases signal-to-noise ratio and is characterized by high sensitivity and high reproducibility of protein peak amplitudes and relative quantitation [42]. Due to the rich representation of host response proteins in the circulating proteome, Deep MALDI spectra from serum and plasma are especially amenable data sources for creating multivariate tests reflecting the immune status of patients that may be associated with response to immunotherapy.

For the purpose of this review we discuss three multivariate proteomic tests created specifically for predicting outcomes from different immune checkpoint inhibitors: the BDX008 test and the immune checkpoint blockade (ICB) test were developed using samples from metastatic melanoma patients, the primary immunotherapy response test (PIR) was developed in an advanced NSCLC population. These tests are based on Deep MALDI spectral acquisition from pretreatment serum samples. Sample preparation, spectral acquisition and spectral processing methods were similar for all three tests and are described in detail in Weber et al., [43]. Although certain elements varied depending on each test’s design goals, several aspects of the machine learning used in test development were common to all three examples. All tests were created using the Diagnostic Cortex^®^ platform [34,35], which incorporates concepts from traditional machine learning and deep learning in a way tailored to work well with small development cohorts with many measured attributes. In particular, the classifiers in each test are hierarchical in structure to allow use of data at multiple levels of abstraction, employ strong regularization via dropout and use ensemble averaging over multiple splits of the development cohort into training and test sets. Hence, reliable evaluations of test performance are obtained from the development set, as demonstrated by subsequent successful independent validation. Each test is composed of one or more binary classifiers generated from this approach. Tests able to stratify patients into more than two classification groups were generated by defining rules for the application of multiple binary classifiers.

The VeriStrat test was developed in a different clinical setting, i.e. for prediction of benefit from epidermal growth factor receptor tyrosine kinase inhibitors (EGFR-TKIs) in NSCLC [44]. The test also utilizes mass spectra from un-processed serum samples; however, it uses conventional MALDI TOF mass spectrometry. The VeriStrat algorithm, a k-nearest neighbors classification scheme based on the relative integrated intensities of eight mass spectral regions, assigns a binary VeriStrat Good (VS Good) or VeriStrat Poor (VS Poor) classification.

The cohorts of patients used in this review are listed in Table 1. More detailed information can be found in the corresponding references. All samples were collected before commencement of treatment under ethics-approved protocols, according to requirements of the corresponding institutions.

## 3. BDX008 Test and ICB Test

BDX008 and the ICB test were developed using the melanoma development set, composed of samples from patients with unresectable melanoma treated with nivolumab in the scope of the NCT01176461 clinical trial [45]. While the same set was utilized in the development of both tests, the ultimate purposes of the two tests were different: BDX008 was designed to provide a simple binary pre-treatment stratification of melanoma patients on anti-PD1 therapy by identifying two subgroups of patients, BDX008+ (good prognosis) and BDX008– (poor prognosis) with significantly different outcomes. The ICB test was designed specifically to identify a smaller subgroup of patients with especially good outcomes: the test assigns a Sensitive (better outcomes) classification to these patients and a Resistant (worse outcomes) classification to the other patients.

BDX008 development was carried out using a single binary classifier trained with 59 features from the mass spectra [52]. The ICB test was developed by pooling the results of seven binary classifiers, each of which was developed using data from a subset of the development cohort. Spectra are classified by each of the seven classifiers. If each classifier returns a good prognosis indicator, the sample is classified as Sensitive. This requirement that samples be classified as good prognosis when assessed by different views of the whole dataset allows identification of a subgroup of patients most likely to have the best outcomes. In total, information from 209 spectral regions is used for the ICB classification [43].

The tests’ performance in the development cohort is summarized in Table 2. Both tests demonstrate a significant separation in outcome between assigned classifications. However, the distribution between good and poor prognosis classifications is different: while 61% of patients are characterized as BDX008+, only 29% are assigned an ICB Sensitive classification. As intended in the test design, a larger proportion of the ICB Sensitive patients reached 2-years survival than BDX008+ patients (67% vs. 55%). In contrast, the proportion of BDX008– patients reaching the 2-year survival landmark was smaller than that of ICB Resistant patients (21% vs. 33%). Similar results are also observed for 3-year survival landmark, Table 2.

Both tests have been validated in multiple independent cohorts of melanoma patients [43,46,53] treated with ICIs; the BDX008 test has also shown potential utility in NSCLC patients treated with nivolumab [50].

## 4. PIR Test

The PIR test was created to stratify outcomes for patients with advanced NSCLC treated with immune checkpoint blockade agents. It was developed using serum samples collected from 116 second line advanced NSCLC patients treated with nivolumab. The test is a hierarchical combination of three binary classifiers, using a total of 274 mass spectral features. The first classifier, similar to BDX008, stratifies the patients into two groups with better and worse outcome. Then two classifiers, each tailored to work well in one of these two subgroups, stratifies the patients further to yield three categories: Sensitive, Intermediate, and Resistant, with better, intermediate, and worse outcomes, respectively. These three classifications can be binarized by combining two out of three categories to differentiate patients with best outcomes (Sensitive) from Not Sensitive (Resistant + Intermediate), or with worst outcomes (Resistant) vs. Not Resistant (Sensitive + Intermediate). In this paper we will concentrate on the latter binarization (Resistant vs. Not Resistant). The PIR test classified 35% of the development set as Resistant; these patients had a significantly shorter overall survival (OS) than the remaining patients (4.3 vs. 11.1 months, *p* = 0.002; HR = 0.48 (95%CI 0.30–0.77)). Similar results were observed in independent cohorts [47]. Notably, when the test was applied to samples from patients treated with the cytotoxic agent docetaxel, Resistant and Not Resistant subgroups had similar OS (*p* = 0.471, HR = 0.80 (95% CI 0.45–1.46) [47], indicating the potential predictive capacity of the test. The predictive ability of a test with respect to treatment is especially important in the clinic, because it allows for the rational selection of therapy depending on pretreatment test classification.

## 5. The VeriStrat Test

The VeriStrat test, originally developed for prediction of benefit from EGFR-TKIs in NSCLC, has been validated in numerous independent studies, demonstrating strong predictive [48,53] and prognostic [54,55,56] properties in various settings. For the purpose of this review we summarize the results of the application of the VeriStrat test to samples from advanced NSCLC patients treated with nivolumab in second and higher lines in the Lung cancer validation set [50], and with various immune checkpoint inhibitors in the scope of the INSIGHT registry study (NCT03289780) [49]. VeriStrat classified 25–39% of patients in these cohorts as VS Poor and demonstrated a significant separation in OS between test classification groups: *p* = 0.039; HR = 0.45 (95%CI 0.21–0.96) in first line monotherapy; *p* < 0.001, HR = 0.32 (95%CI 0.19–0.55) in first line combination of immune checkpoint inhibitors and chemotherapy [49]; and *p* = 0.046, HR = 0.50 (95% CI 0.25–1.00) in the lung cancer validation set treated with nivolumab [50].

## 6. Biological Mechanisms Associated with the Tests 

Possible relationships between test classifications and processes related to the immune status and host response to cancer were explored using protein set enrichment analysis (PSEA), as described previously [51,57]. This set enrichment approach [5] uses a reference set of samples with matched protein panel and mass spectral data for the identification of systematic functional associations of biological processes of interest with test classifications, without prior identification or even specification of individual mass-spectral peaks used in the test. For the purpose of this study we used lung cancer samples from the PSEA reference set, as described in Grigorieva et al. [51].

Table 3 shows the biological processes investigated for correlation with test classifications. Processes with *p* values for association with test classification <0.05 are identified as significant.

The results were consistent with previously published data: Systemic responses of the innate immune system related to acute inflammation, acute phase, and complement activation showed significant associations with the tests studied, with higher levels of these processes in the poor prognosis test classification subgroups [43,51,53]. Components of complement system, acute phase reaction, and acute inflammation are now recognized as a major regulators of cancer development and progression [58,59,60]. Correlations with interferon (IFN) γ signaling and response were common to the BDX008 and ICB tests, while associations with IFN type 1 signaling and response and with innate immune response were significant for both BDX008 and PIR. This is of interest because variations in IFN responses have been linked to limited tumor sensitivity to ICIs [61]. It is also notable that some significant correlations were unique: for example, chronic inflammatory response and extracellular matrix (ECM) organization were significantly associated only with the PIR test, while Type 17 immune response was significantly correlated only with BDX008 (Table 2). There is a big body of data that demonstrates that ECM shapes the inflamed immune microenvironment, plays a vital role at each step of the cancer-immunity cycle], and that alterations of ECM affect treatment outcomes [62,63]. The balance between acute and chronic inflammatory responses, as well as between types 1, 2, and 17 of immune responses, may have opposite effects on anti-cancer immune reactions and on treatment [64,65]. It is intriguing to observe that tests based on the circulating proteome can show different associations with these processes. Hence, in the future they may prove useful for exploring biological mechanisms underlying differences between groups of patients with different test classifications.

## 7. Comparative Characteristics of the Tests

It should be noted that as all tests described above have successfully stratified patients into groups with better and worse prognosis on ICIs, the assignments of good or poor outcome classifications by each test are generally not uncorrelated. There exist subgroups of patients who were classified to either poor or good prognosis groups by all four tests: 18% in the melanoma development set, 22% in the lung cancer development set, and 17% in the PSEA reference set were assigned a poor prognosis; 29%, 16%, and 24% in the respective sets were always classified as good prognosis. Hence, when looking for associations with biological processes of each test individually, we, not surprisingly, found that many biological processes were correlated with all of the tests (Table 3). This similarity can mask the differences in the underlying biology that exist between the tests. To investigate these potential differences, we classified all samples from the melanoma development set, the lung cancer development set, and the PSEA reference set with the BDX008 test, the ICB test, the PIR test, and the VeriStrat test, and examined the correspondence between these test classification for each sample. Further, we analyzed outcomes based on subsetting the samples by pairwise test classifications and used set enrichment analysis to investigate the biological differences between these pairwise classification subsets.

Table 4 shows the distribution of pairwise test classifications for all combinations of two of the four tests (BDX008, ICB, PIR, and VeriStrat), when applied to the melanoma development set, the lung cancer development set, and the PSEA reference set.

**Figure 1:**
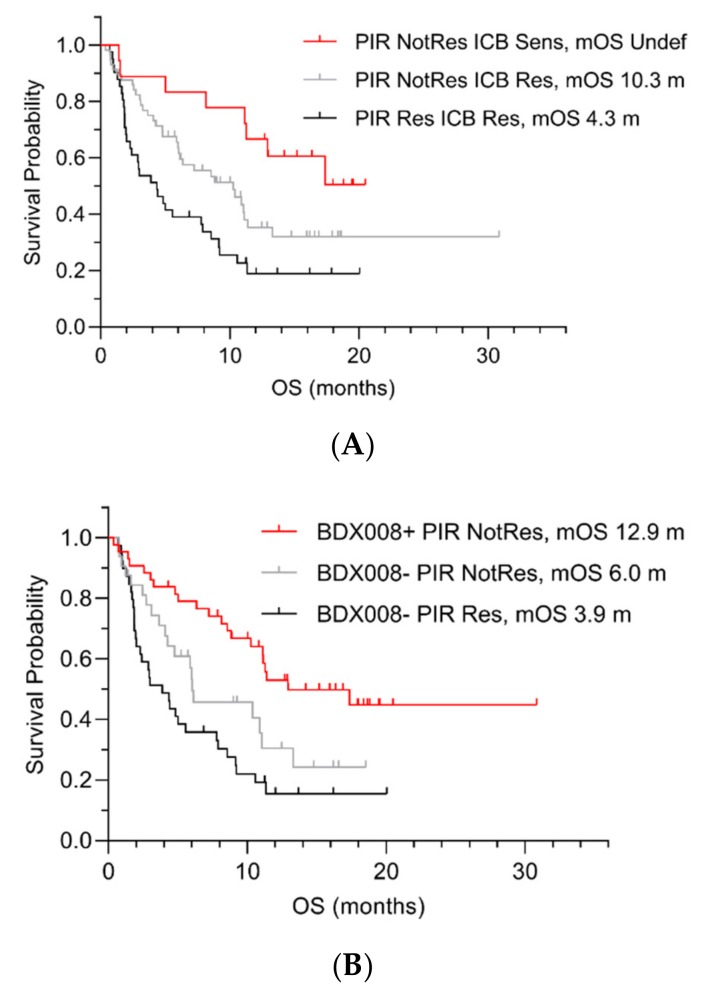

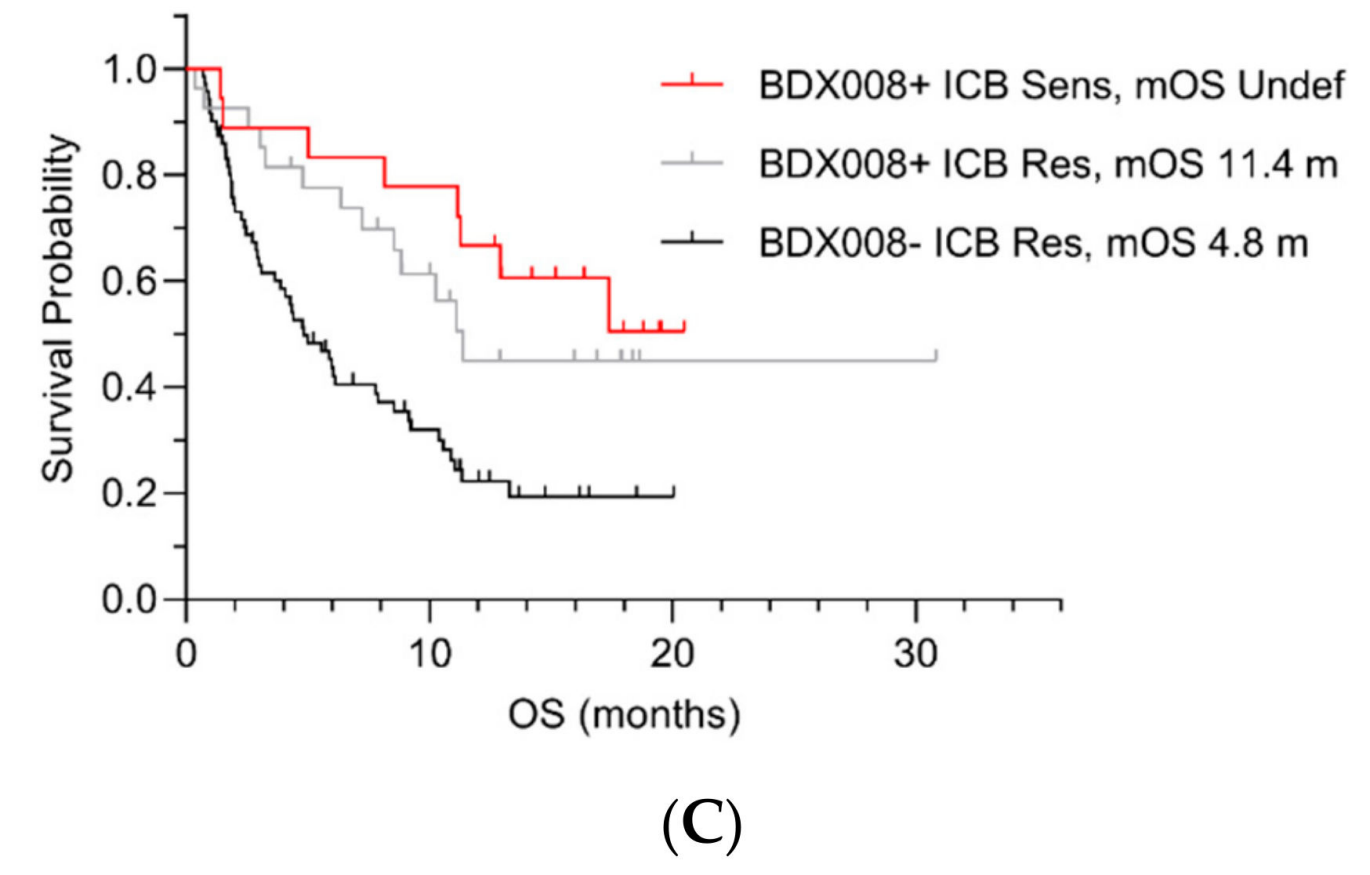
OS by pairwise test classifications in the lung cancer development set by ICB and PIR (**A**), BDX008 and PIR (**B**), and BDX008 and ICB (**C**). Sens = Sensitive; Res = Resistant; NotRes = Not Resistant; mOS = median OS; m = months.

As expected, the distributions between good and poor prognosis groups depended on the test and on the population. For example, both in lung and melanoma sets, a group of ICB Sensitive classifications was a subset of the BDX008+ classification group, while nearly all VS Poor patients had ICB Resistant and PIR Resistant classifications. However, while the majority (91%) of the BDX008– melanoma samples were classified as PIR Resistant, in the lung cancer set BDX008– patients classified as PIR Resistant constituted only 55%. This was an indication of differential performance of the test depending on tumor type. Further comparison was done with respect to outcomes. Figure 1 shows performance of the tests using groupings defined by pairwise classifications of patients in the lung cancer development set.

The results confirmed that the PIR test could further stratify patients with poor prognosis: It separated patients with the ICB Resistant (Figure 1A) and BDX008– classifications (Figure 1B): “PIR Resistant ICB Resistant” patients’ median OS was 4.3 months (Figure 1A); “PIR Resistant BDX008–“ patients had the worst outcomes among all subgroups, with median OS of 3.9 months (Figure 1B). The BDX008 test showed, again, its prognostic power in a lung cancer population treated with ICIs by separating the PIR Not Resistant (Figure 1B) and the ICB Resistant (Figure 1C) patients. Additionally, the ICB test demonstrated potential utility in NSCLC treated with ICIs by stratifying outcome in patients classified as PIR Not Resistant or BDX008+. The median OS in the groups with the “ICB Sensitive PIR Not Resistant” and the “ICB Sensitive BDX008+” classifications was not reached, while in the “PIR Not Resistant ICB Resistant” subgroup it was 10.3 months and in the “BDX008+ ICB Resistant” subgroup it was 11.4 months (Figure 1A,C, respectively).

To shed more light on underlying mechanistic differences between the tests, we applied the PSEA approach to evaluate associations of biological processes with splits of the test classification groups into the pairwise subsets. For this purpose, we applied BDX008, ICB, PIR, and VeriStrat tests to the PSEA reference set. The results are summarized in Table 5. Similar to the results in Table 3, when used to divide subgroups generated by other tests, BDX008, ICB, and VeriStrat showed a pronounced association with many common biological processes, such as acute phase reaction, complement activation, and immune tolerance and suppression (with increased levels of these processes in poor prognosis test classification subgroups). Type 17 immune response, in addition to being important to the BDX008 classification, also showed a significant association with VeriStrat classification. Interestingly, refinement of test groups by PIR classification was consistently associated with wound healing and innate immune response, while the other innate immunity processes, that showed associations between PIR resistant and PIR Not resistant within the whole population (Table 2), did not retain their significance (Table 5).

Though the data in Figure 1A–C were obtained using a different set of lung cancer patients than the PSEA reference set analyzed in Table 5, taken together, they may provide some preliminary evidence in the context of the effects on outcomes. For example, it is intriguing that separation of patients in the subgroups classified as ICB Resistant or as BDX008– by the PIR test (Figure 1A,B, respectively) might be a result of the difference in wound healing as an underlying mechanism. It would also be interesting to gain more understanding of the role of Type 1 immune response that was significantly correlated with separation of the BDX008+ subset of patients by the ICB tests (Figure 1C). Evaluation of differential effects of various aspects of innate immunity in subgroups of patients defined by different tests may potentially lead to new insights into tumor biology and the origins of sensitivity and resistance to treatment.

## 8. Independence of Proteomic Tests from Clinical Characteristics and Biomarkers for Immunotherapy

Until recently it was generally accepted that the course of the disease and outcomes of treatment could be predicted from the properties of the tumor and its immediate microenvironment. Hence, it was important to check that serum-based host response proteomic tests provided new, clinically relevant information, in addition to tissue-based biomarkers and standard clinical prognostic factors.

Data from multiple studies of the VeriStrat test with patients receiving targeted therapies or chemotherapy have indicated association of the serum proteomic test classification with some tumor-related biomarkers (such as presence of sensitizing EGFR mutations), but not all of them (no association has been found with KRAS mutational status). However, even when associations have been identified, the test remained a significant predictor of outcomes in multivariate analysis and was able to stratify outcomes within tumor biomarker defined subgroups [66]. In the case of immunotherapies, the most relevant tumor/TME biomarker is PD-L1 expression. No significant correlation has been observed between PD-L1 expression and classifications for the tests reviewed here: in the melanoma development set the *p*-values of association between test classifications and PDL 1 >5% vs. <5% was 0.693 for the BDX008 test [52] and 0.704 for the ICB test [43]. There was no indication of correlation of the PIR test classifications with PD-L1 expression (cut-off 1%) in the lung cancer development cohort [47]. Association of VeriStrat classification with PD-L1 status did not reach statistical significance in the interim analysis of the INSIGHT study (*p* = 0.119) and continued to predict outcome when adjusted for PD-L1 status in multivariate analysis [49].

Tests classifications were found to be correlated with several prognostic factors: BDX008– classification was significantly associated with higher levels of LDH (Fisher’s test *p* = 0.006) and NLR > 5 (Fisher’s test *p* = 0.003) in melanoma patients [46]; the ICB test correlated with LDH (*p* = 0.006) and with baseline tumor size (*p* < 0.001) [43]. However, all the tests retained their statistical significance for prediction of OS in multivariate analyses that included various prognostic factors and available biomarker measurements: the BDX008 test was an independent predictive factor (*p* = 0.009) when adjusted for BRAF mutation status, line of treatment, and LDH in an independent validation cohort of melanoma patients [46]; the ICB classification remained a significant predictor of survival (*p* = 0.002) when adjusted for gender, age, LDH, PD-L1 expression, prior treatment, and tumor size [43]. In the lung cancer setting, the PIR test (Resistant vs. Not Resistant) was a significant predictor of survival when adjusted for ECOG performance status, smoking history, histology, and PD-L1 expression (*p* = 0.030) [67]. VeriStrat classification, when analyzed in a cohort of patients treated with immunotherapy-containing regimens in the INSIGHT study was significantly associated with survival (*p* < 0.001) when adjusted for multiple factors, including PD-L1 expression, ECOG performance status, and type of immunotherapy regimen [49].

**Figure 2:**
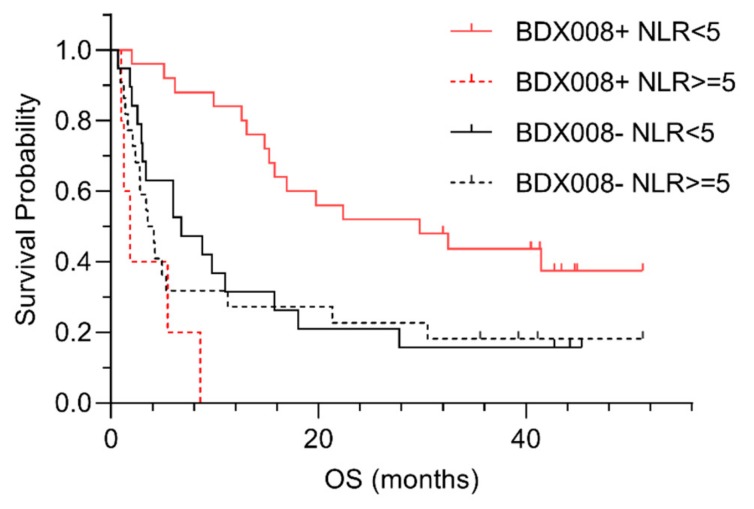
Kaplan–Meier plots of outcome data by BDX008 classification for patients in defined by neutrophil-to-lymphocyte ratio (NLR).

In the case of melanoma patients, we could evaluate the potential interaction between test classifications and NLR [23]—a biomarker of systemic inflammation that has previously shown association with outcomes on ICIs [24,25]. The Kaplan–Meier plots suggested that the difference in outcomes between BDX008 classifications may be qualitatively different in the subgroups defined by NLR: it appeared that patients with NLR <5 classified as BDX008+ had especially good outcomes on anti-PD-1 treatment; while at the same time patients classified as BDX008– had poor prognosis independently of NLR status [46] (Figure 2).

A Cox proportional hazard analysis of the interaction between BDX008 classification and NLR resulted in an interaction *p* = 0.002, confirming the importance of both variables for prognosis [46]. A significant interaction between BDX008 and NLR is notable, because it shows that information about host response that is associated with the BDX008 test is complementary to the measurements of systemic inflammation that are based on immune cell counts in blood.

## 9. Discussion

With the acknowledgment of the essential role of the immune system in cancer and of inflammation as one of the enabling characteristics of cancer [68,69], it has become clear that measuring only tumor-related characteristics cannot be sufficient for the adequate prediction of the course of the disease and response to treatment. Efforts have been aimed at creating multivariate biomarkers that reflect broader biological interactions and inflammatory responses. For example, an 18-gene tumor inflammation signature (TIS), measuring pre-existing, adaptive, intra-tumoral immune response, was shown to enrich for patients benefiting from pembrolizumab, while being only marginally prognostic without treatment. The TIS scores were minimally correlated with TMB, and the expression patterns of the TIS were conserved in different tumors, allowing it to serve as a pan-cancer measurement of the inflamed tumor phenotype [70]. Genes in the TIS signature reflected the biology of antigen presentation, T cell and NK cell abundance, interferon activity and markers of T cell exhaustion. Another recently reported microarray DNA methylation signature (EPIMMUNE) showed promising results in lung cancer, specifically for treatment with PD-1 blockade. This signature was also not associated with PD-L1 expression, the presence of CD8+ cells, or mutational load [71]. Innate resistance to anti-PD-1 treatment was found to be associated with a transcriptional signature referred to as IPRES, indicating concurrent upregulation of genes involved in mesenchymal transition, cell adhesion, extracellular matrix remodeling, angiogenesis, and wound healing [72]. However, the results of the IPRES study highlighted one of the potential limitations of all gene expression profile analyses: Spatial localization of immunocytes and expression of markers on their surface are perhaps more critical in their architectural arrangement within the tumor microenvironment than their absolute levels [73]. In contrast, the circulating proteome reflects the state of the whole organism and adds a new dimension to such gene expression-based measurements.

The importance of host response to the disease that is captured in the circulating proteome came into focus relatively recently. The results presented herein provide strong evidence that tests based on measurements of serum and plasma contain information complementary to other biomarkers. Notably, the tests discussed in this paper were validated in multiple independent cohorts, providing convincing evidence for their adequate performance in various settings. Moreover, the results for PIR test indicated potential predictive properties of the test and provided evidence that it is likely not merely prognostic, i.e., reflective of the physiological state of the organism independent of subsequent interventions, but may be specific to therapy. The significance of these results is enhanced by the unsatisfactory performance of the currently used biomarkers. Application of set enrichment analysis allowed us to elucidate the biological underpinnings of the tests, demonstrating their meaningful correlations with innate immune system responses, that have previously been implicated in cancer development and treatment-related outcomes by other methods. Certainly, our ability to identify underlying biological mechanisms has limitations that are related to the sensitivity of mass-spectral methods and are inherent to the set enrichment approach discussed in detail in Grigorieva et al. [51]. Deeper understanding of the biological processes underlying proteomic tests would require additional experimental testing of the proposed mechanisms.

In terms of methodology, the results illustrate the advantages of applying modern machine learning techniques to datasets with complex information content. We have demonstrated that, depending on the specific question at hand, different approaches to test design result in different tests, which are optimized for a specific performance goal. Even using only one machine learning algorithm, tests can be optimized for target performance in a specific indication by changing the development cohorts to match the indication (e.g., NSCLC patients for PIR versus melanoma patients for BDX008 and ICB) or by constructing tests from different hierarchical arrangements of classifiers (e.g., single binary classifier trained on the whole development cohort for BDX008 versus pooling of results over seven binary classifiers each training on a subset of the development cohort for ICB). We have also demonstrated that tests developed with the same type of samples (serum) and the same measurement technology (Deep MALDI), but using different algorithmic approaches and sample sets, may leverage different aspects of host response to the tumor. Consequently, application of more than one test to the same sample may result in further refinement of a patient population into categories receiving either particular high or low benefit from therapy. Independent validation of any proposed combination of tests would be required to conclusively demonstrate improved patient stratification performance in a specific indication.

## 10. Conclusions

Combining information from orthogonal tumor-based and host response biomarkers seems to be the most promising avenue for integrating multifaceted aspects of interactions between cancer and the immune system that define outcomes on immune therapies. Multivariate proteomic serum-based tests have demonstrated good performance and were validated in various indications in multiple independent studies. Mechanisms associated with these tests are related to important systemic aspects of tumor–host interaction. Taking into account their utility and their ability to complement other biomarkers, these tests should be added to the arsenal of tools used in broad clinical practice.

## Figures and Tables

**Table 1:** Cohorts of patients.

Cohort	Patients (pts)	N pts	Reference
Melanoma development set	Unresectable melanoma patients treated with nivolumab in the NCT01176461 clinical trial (74% prior ipilimumab therapy)	119	Weber et al., 2013 [45]
Melanoma validation set	Unresectable melanoma patients treated with nivolumab or pembrolizumab in 2nd and higher lines (35% prior targeted therapy)	71	Ascierto et al., 2019 [46]
PIR development set	NSCLC patients treated with nivolumab in 2nd line (prior platinum-based chemotherapy)	116	Aerts et al. 2018 [47]
PIR control set	NSCLC patients treated with docetaxel in 2nd line (prior platinum-based chemotherapy)	68	Gregorc et al., 2014 [48]
INSIGHT validation sets	NSCLC patients treated with ICI in scope of INSIGHT registry study (NCT03289780)		Rich et al., 2019 [49]
	Monotherapy ICI 1st line	46	
	ICI plus chemotherapy 1st line	33	
Lung cancer validation set	NSCLC patients treated with nivolumab in 2nd and higher lines enrolled in a single-institutional translational research study (prior platinum-based chemotherapy)	60	Grossi et al., 2017 [50]
PSEA reference set	NSCLC patients; samples obtained from commercial biobanks Conversant Bio (Huntsville, AL) and Oncology Metrix (Fort Worth, TX)	100	Grigorieva et al., 2019 [51]

**Table 2:** Performance of BDX008 and immune checkpoint blockade (ICB) tests in development cohort.

Test	BDX008		ICB	
Classification	BDX008+	BDX008–	ICB Sensitive	ICB Resistant
*n* (%)	72 (61%)	47 (39%)	34 (29%)	85 (71%)
2-year survival	55%	21%	67%	33%
3-year survival	51%	14%	58%	28%
OS curves comparison	HR = 0.38 (0.19–0.55), *p* < 0.001		HR = 0.37 (0.19–0.71), *p* = 0.002	

OS: Overall survival. HR: hazard ratio.

**Table 3:** Associations between proteomic tests and biological processes.

Biological Processes	BDX008	ICB	PIR (Resistant /Not Resistant)	VeriStrat
Acute inflammatory response	x	x	x	x
Acute phase reaction	x	x	x	x
Angiogenesis				
B cell-mediated immunity				
Chronic inflammatory response			x	
Complement activation	x	x	x	x
Cytokine production in immune response				
Epithelial-mesenchymal transition				
Extracellular matrix organization			x	
Glycolysis activation				
IFN type 1 signaling/response	x		x	
IFN γ signaling/response	x	x		
Immune tolerance/suppression	x	x		x
Innate immune response	x		x	
NK cell-meditated immunity				
Response to hypoxia				
T cell-mediated immunity				
Type 1 immune response				
Type 17 immune response	x			
Type 2 immune response				
Wound healing	x		x	

Significant associations indicated by x.

**Table 4:** Distribution of number of patients with pairwise classification assignments by sample set for all pairs of the four tests.

Test 1	Test 2	Melanoma Development set			Lung Cancer Development Set			PSEA Reference Set		
	**BDX008**	BDX008+	BDX008–	Total	BDX008+	BDX008–	Total	BDX008+	BDX008–	Total
**ICB**	Sensitive	34	0	34	18	0	18	24	0	24
	Resistant	38	47	85	27	71	98	19	57	76
	Total	72	47	119	45	71	116	43	57	100
	**BDX008**	BDX008+	BDX008–	Total	BDX008+	BDX008–	Total	BDX008+	BDX008–	Total
**PIR**	NotResist	64	4	68	43	32	75	42	28	70
	Resistant	8	43	51	2	39	41	1	29	30
	Total	72	47	119	45	71	116	43	57	100
	**BDX008**	BDX008+	BDX008–	Total	BDX008+	BDX008–	Total	BDX008+	BDX008–	Total
**VeriStrat**	VS Good	72	0	72	45	0	45	43	35	78
	VS Poor	26	21	47	43	28	71	0	22	22
	Total	98	21	119	88	28	116	43	57	100
	**ICB**	Sensitive	Resistant	Total	Sensitive	Resistant	Total	Sensitive	Resistant	Total
**PIR**	NotResist	34	34	68	18	57	75	23	47	70
	Resistant	0	51	51	0	41	41	1	29	30
	Total	34	85	119	18	98	116	24	76	100
	**ICB**	Sensitive	Resistant	Total	Sensitive	Resistant	Total	Sensitive	Resistant	Total
**VeriStrat**	VS Good	34	64	98	18	70	88	24	54	78
	VS Poor	0	21	21	0	28	28	0	22	22
	Total	34	85	119	18	98	116	24	76	100
	**VeriStrat**	VS Good	VS Poor	Total	VS Good	VS Poor	Total	VS Good	VS Poor	Total
**PIR**	NotResist	68	0	68	56	3	59	64	6	70
	Resistant	30	21	51	32	25	57	14	16	30
	Total	98	21	119	88	28	116	78	22	100

NotResist: Not Resistant.

**Table 5:** Processes associated with the pairwise test stratifications.

#	Test Stratification	Subgroup (*n*)	Process	*p*
1	PIR Not resistant/resistant	BDX008– (57)	Wound healing	0.048
		ICB Resistant (76)	Innate immune response	0.012
			Wound healing	0.013
		VeriStrat Good (78)	Innate immune response	0.011
			Wound healing	0.014
2	ICB sensitive/resistant	BDX008+ (43)	Complement	0.026 *
			Type 1 immune response	0.049 *
		PIR Not Resistant (70)	Complement	<0.001
			Acute inflammatory response	<0.001
			Acute phase reaction	0.001
			Immune tolerance/suppression	0.007
			IFN γ signaling/response	0.011
3	BDX008 +/−	ICB Resistant (76)	Acute phase reaction	<0.001
			Innate immune response	0.031
			Acute inflammatory response	0.031
			Type 17 immune response	0.032
			IFN γ signaling/response	0.049
		PIR Not Resistant (70)	Acute phase reaction	<0.001
			IFN γ signaling/response	0.001
			Acute inflammatory response	0.003
			Immune tolerance/suppression	0.013
			Type 17 immune response	0.013
			Complement	0.020
4	VeriStrat (VSG/VSP)	PIR Not Resistant (70)	Immune tolerance/suppression	<0.001 *
			Acute phase reaction	0.001 *
			Type 17 immune response	0.007 *
			Complement	0.008 *

* *p*-values calculated using the protein set enrichment analysis (PSEA) method with the standard enrichment score (ES) definition in Subramanian et al. [5] rather than the ES defined averaged over 25 splits of the dataset defined in Roder et al. [59].
